# Supplementary material for: Standardizing workflows in imaging transcriptomics with the abagen toolbox
Source: eLife. 2021 Nov 16;10:e72129. doi: 10.7554/eLife.72129 (PMC8660024; doi:10.7554/eLife.72129)
Supplement: Supplementary file 1. — The default settings for the 17 processing steps considered when processing the AHBA data with abagen. An entry of ‘—' indicates that this is a required, user-supplied parameter. A blank entry indicates that the processing step is not implemented by default. Refer to Table 1 and Methods: Gene expression pipelines for further details. [file elife-72129-supp1.pdf]

Table S1. **Default `abagen` pipeline options |** The default settings for the 17 processing steps considered when processing the AHBA data with `abagen`. An entry of "—" indicates that this is a required, user-supplied parameter. A blank entry indicates that the processing step is not implemented by default. Refer to Table **??** and *Methods: Gene expression pipelines* for further details.

Option	*Default*
Volumetric or surface atlas	—
Individualized or group atlas	—
Use non-linear MNI coordinates	True
Mirror samples across L/R hemisphere	
Update probe-to-gene annotations	True
Intensity-based filtering threshold	50%
Inter-areal similarity threshold	
Probe selection method	differential stability
Donor-specific probe selection	aggregate
Missing data method	
Sample-to-region matching tolerance	2mm
Sample normalization method	scaled robust sigmoid
Gene normalization method	scaled robust sigmoid
Normalize only matched samples	True
Normalizing discrete structures	False
Sample-to-region combination method	donors
Sample-to-region combination metric	mean
